# Assessment of Genotype Imputation Performance Using 1000 Genomes in African American Studies

**DOI:** 10.1371/journal.pone.0050610

**Published:** 2012-11-30

**Authors:** Dana B. Hancock, Joshua L. Levy, Nathan C. Gaddis, Laura J. Bierut, Nancy L. Saccone, Grier P. Page, Eric O. Johnson

**Affiliations:** 1 Behavioral Health Epidemiology Program, Research Triangle Institute International, Research Triangle Park, North Carolina, United States of America; 2 Research Computing Division, Research Triangle Institute International, Research Triangle Park, North Carolina, United States of America; 3 Department of Psychiatry, Washington University, St. Louis, Missouri, United States of America; 4 Department of Genetics, Washington University, St. Louis, Missouri, United States of America; 5 Genomics, Statistical Genetics, and Environmental Research Program, Research Triangle Institute International, Atlanta, Georgia, United States of America; National Institute of Environmental and Health Sciences, United States of America

## Abstract

Genotype imputation, used in genome-wide association studies to expand coverage of single nucleotide polymorphisms (SNPs), has performed poorly in African Americans compared to less admixed populations. Overall, imputation has typically relied on HapMap reference haplotype panels from Africans (YRI), European Americans (CEU), and Asians (CHB/JPT). The 1000 Genomes project offers a wider range of reference populations, such as African Americans (ASW), but their imputation performance has had limited evaluation. Using 595 African Americans genotyped on Illumina’s HumanHap550v3 BeadChip, we compared imputation results from four software programs (IMPUTE2, BEAGLE, MaCH, and MaCH-Admix) and three reference panels consisting of different combinations of 1000 Genomes populations (February 2012 release): (1) 3 specifically selected populations (YRI, CEU, and ASW); (2) 8 populations of diverse African (AFR) or European (AFR) descent; and (3) all 14 available populations (ALL). Based on chromosome 22, we calculated three performance metrics: (1) concordance (percentage of masked genotyped SNPs with imputed and true genotype agreement); (2) imputation quality score (IQS; concordance adjusted for chance agreement, which is particularly informative for low minor allele frequency [MAF] SNPs); and (3) average r2hat (estimated correlation between the imputed and true genotypes, for all imputed SNPs). Across the reference panels, IMPUTE2 and MaCH had the highest concordance (91%–93%), but IMPUTE2 had the highest IQS (81%–83%) and average r2hat (0.68 using YRI+ASW+CEU, 0.62 using AFR+EUR, and 0.55 using ALL). Imputation quality for most programs was reduced by the addition of more distantly related reference populations, due entirely to the introduction of low frequency SNPs (MAF≤2%) that are monomorphic in the more closely related panels. While imputation was optimized by using IMPUTE2 with reference to the ALL panel (average r2hat = 0.86 for SNPs with MAF>2%), use of the ALL panel for African American studies requires careful interpretation of the population specificity and imputation quality of low frequency SNPs.

## Introduction

Genotype imputation is often conducted in genome-wide association studies (GWAS) as an efficient approach to expand coverage of single nucleotide polymorphisms (SNPs), enabling meta-analysis of GWAS from different genotyping platforms [1] and fine-mapping in regions of interest to identify potentially causal variants [2]. To infer untyped SNP genotypes, imputation uses reference haplotype panels genotyped at a dense set of SNPs, historically from the original HapMap [3], [4] populations of Yorubas from West Africa (YRI), European Americans (CEU), northern Chinese (CHB), and Japanese (JPT). CEU serves as a good reference for genotype imputation in European or European American studies, and CHB and JPT serve as good references for East Asian studies [5].

Genotype imputation in admixed populations has not performed as well, because their genetic diversity is greater than the original reference populations [6]. In African Americans, combining YRI with at least one other reference population boosts imputation performance when compared to YRI alone [5], [7], [8], but an optimal imputation strategy is not well established. Two or more reference populations can be combined in their entirety [9], [10], combined in equal proportions [11], [12], or weighted to match the ancestral proportions of the study population [13], [14]. Alternatively, the imputation procedure can be conducted sequentially (once for each selected reference population) rather than as a combined population, followed by merging the imputed genotypes [15], [16]. Most recently, advancements in imputation software have been developed to create tailored reference panels for individual study subjects using all available reference populations as a starting point [17]. This type of approach is particularly attractive for admixed populations and is promoted for its simplistic and practical use for investigators.

Imputation in African Americans has typically relied on some composition of the YRI, CEU, and CHB+JPT populations from HapMap [3], [4]. The 1000 Genomes project [18] now offers a wider range of reference populations that provide a better match of allele frequencies and linkage disequilibrium patterns for admixed populations, such as African Americans from the Southwest United States (ASW). However, there has been limited evaluation of the imputation performance of the newer reference populations in admixed study populations [8]. The present study offers a thorough evaluation of SNP genotype imputation performance in African Americans, by comparing imputation results using four different imputation software programs (IMPUTE2, BEAGLE, MaCH, and MaCH-Admix) and three reference panels consisting of different combinations of 1000 Genomes (February 2012 release) populations that are either closely or more broadly related to African Americans.

## Methods

### Study Subjects, Genotyping, and Quality Control

We obtained genome-wide genotyping data available from Illumina’s iControl database (iControlDB from Illumina, Inc., San Diego, CA) for 830 African Americans genotyped for 561,466 SNPs on the Illumina HumanHap550v3 BeadChip. Data were downloaded on January 19, 2011. Quality control (QC) was implemented on SNPs and subjects using PLINK [19] unless otherwise stated. The QC criteria were selected to resemble standard criteria used for GWAS. Genotyped SNPs were excluded due to minor allele frequency (MAF) <1%, call rate<95%, or Hardy-Weinberg equilibrium (HWE) *p*-value<0.0001. There remained 541,860 autosomal SNPs (96.5%), of which 8,101 SNPs from chromosome 22 were used for imputation.

The subsequent subject-level QC procedures are outlined in Figure S1. First, we verified that all iControlDB African Americans had call rates>95%. Identity-by-state (IBS) estimates were then calculated to identify possibly duplicated subjects. For pairs of subjects having IBS>99%, we retained the subjects with the highest call rate from each pair. Identity-by-descent (IBD) estimates are often generated from GWAS data to remove subjects with cryptic relatedness, whose inclusion would violate independence assumptions in subsequent statistical analyses. However, population stratification may inflate these estimates, so we used the KING program, which was designed to circumvent the inflation of IBD estimates due to stratification [20]. We used a kinship coefficient threshold of 0.0441 to identify clusters of third-degree or closer relatives [20], and we retained only one subject having the highest call rate from each relative cluster. Following the aforementioned QC criteria, 774 (93.3%) subjects remained. None of these subjects had excessive homozygosity or discordance between reported gender and estimated gender based on chromosome X SNP data.

The iControlDB subjects were then evaluated for population structure to identify ancestral outliers. We implemented the pairwise population concordance test in PLINK, which is based on the observed proportion of IBS loci pairs, and we identified eight subjects who were significantly different (P<0.0005) from 95% of the rest of the population. The ancestral outliers were confirmed using the STRUCTURE program [21] and were excluded from further analyses. Ancestral proportions of all iControlDB subjects were inferred via comparison to HapMap CEU, YRI, CHB, and ASW populations in STRUCTURE. As shown in Figure S2, the resulting ancestral proportions indicate that the identified ancestral outliers have relatively high Asian ancestry (up to 78%) or European ancestry (up to 98%). Given that the minimum African ancestral proportion among ASW subjects was 52.7%, we excluded 179 subjects with an African ancestry less than 60%. A list of subjects excluded due to ancestral misclassification is provided in Table S1. The final analysis dataset included 595 (71.7%) subjects.

### Reference Haplotype Panels

We obtained prephased reference haplotypes from the February 2012 release of the 1000 Genomes project (ftp://ftp.1000genomes.ebi.ac.uk/vol1/ftp/release/20110521/, accessed March 7, 2012). We created three reference haplotype panels based on different combinations of 1000 Genomes populations. The first 1000 Genomes reference panel was created by combining YRI (N = 88), CEU (N = 85), and ASW (N = 61) populations, which were specifically chosen to provide a close match to African American study populations. We previously evaluated several different combinations of HapMap phase III reference populations for our African American study population and found that the combined YRI+CEU+ASW panel had optimal imputation performance [22]. The YRI+CEU+ASW panel has also been shown elsewhere to outperform other combined HapMap phase III panels [8].

The second 1000 Genomes reference panel, having eight populations of African (AFR) or European (EUR) descent, was chosen as a broader match for African Americans. The combined AFR+EUR panel included YRI, CEU, ASW, as well as Kenyans (LWK, N = 97), Finns (FIN, N = 93), Britons (GBR, N = 89), Spaniards (IBS, N = 14), and Italians (TSI, N = 98). Additional comparisons of the YRI+CEU+ASW and AFR+EUR reference panels were made using HapMap phase III (http://hapmap.ncbi.nlm.nih.gov/downloads/phasing/2009-02_phaseIII/HapMap3_r2, accessed January 25, 2011) and the August 2010 release of 1000 Genomes (ftp://ftp.1000genomes.ebi.ac.uk/vol1/ftp/release/20100804/, accessed June 17, 2011), as shown in Figures S3, S4, S5.

The third, most diverse 1000 Genomes reference panel used all 14 available populations from the February 2012 release of 1000 Genomes (YRI, CEU, ASW, LWK, FIN, GBR, IBS, TSI, Columbians [CLM, N = 60], Mexican Americans [MXL, N = 66], Puerto Ricans [PUR, N = 55], Northern Chinese [CHB, N = 97], Southern Chinese [CHS, N = 100], and Japanese [JPT, N = 89]). This “cosmopolitan” approach of using all available reference populations has been advocated by others, particularly for diverse study populations [2], [5], [17], [23].

### Imputation

As reviewed elsewhere [2], there are several software packages with different algorithms available for genotype imputation. We compared imputation results using four widely used programs: IMPUTE2 version 2.2.2 [17], BEAGLE version 3.3 [24], MaCH version 1.0.16.c [5], and MaCH-Admix (beta version 2.0.150). MaCH and BEAGLE were previously shown to outperform other software programs not evaluated here [25], while MaCH-Admix and IMPUTE2 represent recent software advancements that create tailored reference panels for individual study subjects. It is important to note that the current version of MaCH-Admix is a prerelease. MaCH-Admix is being developed to extend the capabilities and shorten the computing run time of MaCH 1.0, by incorporating a novel piecewise reference selection method that creates the tailored reference panels. All of the available details on the Mach-Admix beta version can be found at http://www.sph.umich.edu/csg/yli/MaCH-Admix/.

Imputation using IMPUTE2 [17] was preceded by prephasing the study genotypes with the ShapeIT program to estimate haplotypes, using 500 conditioning states, an effective population size of 17,469 as recommended for populations of African descent, and default settings for all other program options. The estimated study haplotypes were then input into IMPUTE2 to impute SNPs available on the reference haplotype panel. Imputations were done on 4.5 MB chunks with 1 MB flanking buffers. Default options were used, except that k_hap was set to the number of haplotypes in each reference panel. Smaller values of k_hap would likely produce similar accuracy at lower computational cost [17].

Imputation using BEAGLE [24] was conducted using all default options. When using MaCH [5], model parameters (crossover and error rates) were estimated prior to imputation by randomly selecting a subset of 200 haplotypes from the study subjects and running 100 iterations with the command options –compact and –greedy. Genotype imputation was then carried out using the model parameter estimates from the previous round with command options of –compact, –greedy, –mle, and –mledetails specified. When using MaCH-Admix, the step 1 imputation settings included: –runmode EstimateParameterOnly -r 30, –fittingstates 200, and –autoflip. MaCH-Admix step 2 setting included: –runMode ImputeOnly, –compact, and –autoflip.

For each imputed SNP, we obtained a genotype dosage value (a fractional value between 0 and 2 indicating the expected number of minor allele copies) and the most likely discrete genotype, either directly from the output of the imputation software (MaCH, MaCH-Admix, and BEAGLE) or via conversion of the software output (IMPUTE2). For all imputation procedures, we masked 2% of the genotyped SNPs to allow direct comparisons of their true and imputed genotypes. The imputation procedures were repeated 10 times with 10 different sets of randomly masked SNPs, with one exception due to excessive computational runtime (MaCH using the ALL panel, for which only one imputation procedure is presented). Indel variants from 1000 Genomes panels were not included, in order to focus on evaluating the performance of SNP genotype imputation.

### Imputation Performance Metrics

For each imputation scenario based on the different software programs and reference panels, we calculated three imputation performance metrics, which captured different features of imputation accuracy and quality. First, after masking 2% of the genotyped SNPs, we calculated the concordance rate as the percentage of genotype calls for which the true genotype matches the most likely discrete imputed genotype. This concordance rate calculation, based on discrete imputed SNP genotypes, has been used often as a measure of imputation accuracy [6], [15], [25]–[29]. Second, using the same masked SNPs, we calculated the imputation quality score (IQS) as previously described by Lin et al. to adjust the concordance rate for chance agreement between imputed and true genotypes [30]. More specifically, the IQS, which is partly motivated by Cohen’s kappa statistic to quantify interrater agreement [31], controls for allele frequencies by taking the observed agreement between imputed and true genotypes (i.e., concordance rate) and subtracting out chance agreement, based on the sum of products of marginal frequencies that would occur if genotypes were called at random [30]. Therefore, the IQS metric is particularly useful for evaluating imputation accuracy of low frequency SNPs. Concordance and IQS results were averaged across all masked SNPs and then averaged across the 10 different sets of randomly masked SNPs. Third, we calculated r2hat (estimated squared correlation between each imputed genotype and its true underlying genotype) using the genotype dosage values and then averaged the r2hat values across all polymorphic imputed SNPs. Each software program generates its own imputation quality metric for each SNP (info for IMPUTE2, allelic r2 for BEAGLE, and r2 for MaCH and MaCH-Admix), as reviewed by Marchini and Howie [2]. The program-specific metrics are highly correlated [2], but because they are different in character, calculation of r2hat (script available at http://www.sph.umich.edu/csg/yli/software.html) was needed to generate a single, common metric to assess imputation quality across the programs.

Given the need for computational efficiency with the large number of imputations (10 repetitions for each imputation procedure), our analyses focused on chromosome 22, as other studies evaluating imputation performance have done [15], [29], [32]. To ensure comparability of imputation results across chromosomes, we conducted imputation using MaCH on chromosomes 1 and 22 with reference to YRI from HapMap phase III. Imputation performance was slightly better on chromosome 1 (concordance = 92.1% and average r2 = 0.83) than on chromosome 22 (concordance = 89.2% and average r2 = 0.80), likely due to a larger number of SNPs available for comparison and slightly higher linkage disequilibrium levels on the larger chromosome.

## Results

**Figure 1:**
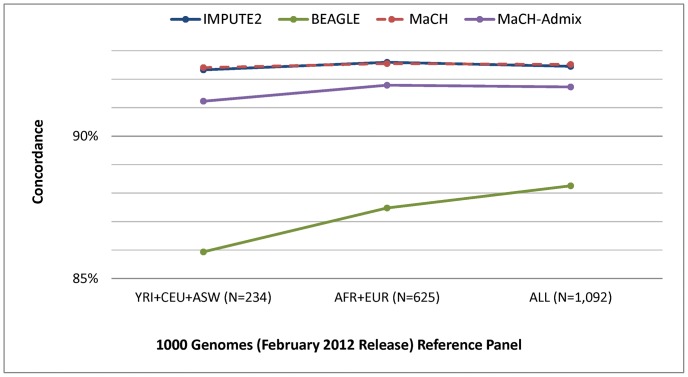
Concordance resulting from four different imputation programs and three different 1000 Genomes (February 2012 release) reference panels. Concordance rates were based on masking 2% of the genotyped SNPs on chromosome 22 and comparing imputed and true genotypes. The number of subjects corresponding to each reference panel is shown in parentheses.

**Figure 2:**
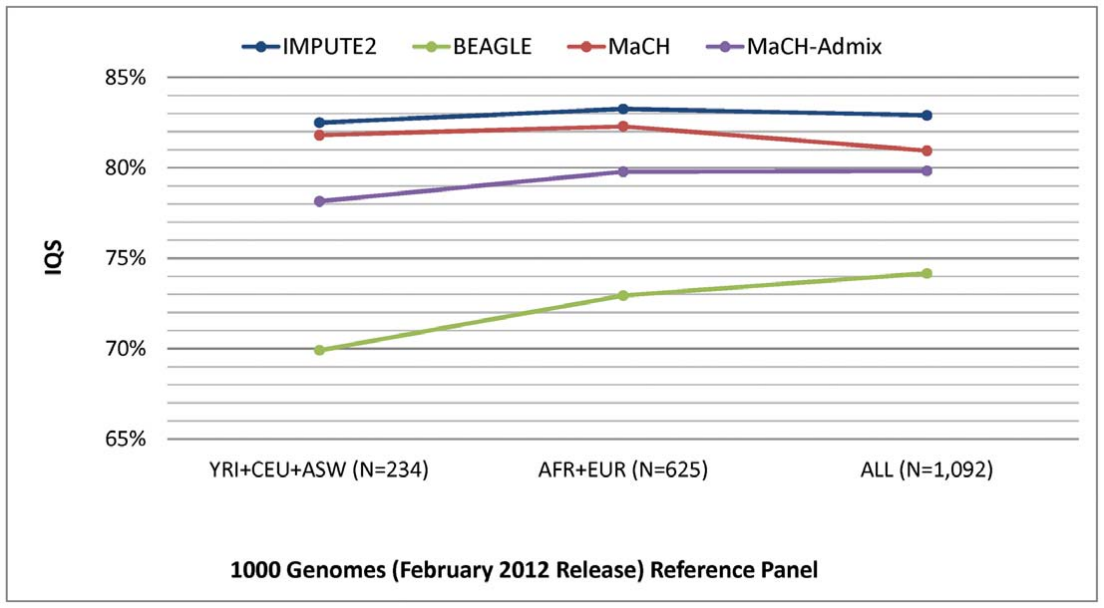
Imputation quality score (IQS) resulting from four different imputation programs and three different 1000 Genomes (February 2012) reference panels. IQS results were based on masking 2% of the genotyped SNPs and adjusting the concordance rate chance agreement between imputed and true genotypes. The number of subjects corresponding to each reference panel is shown in parentheses.

**Figure 3:**
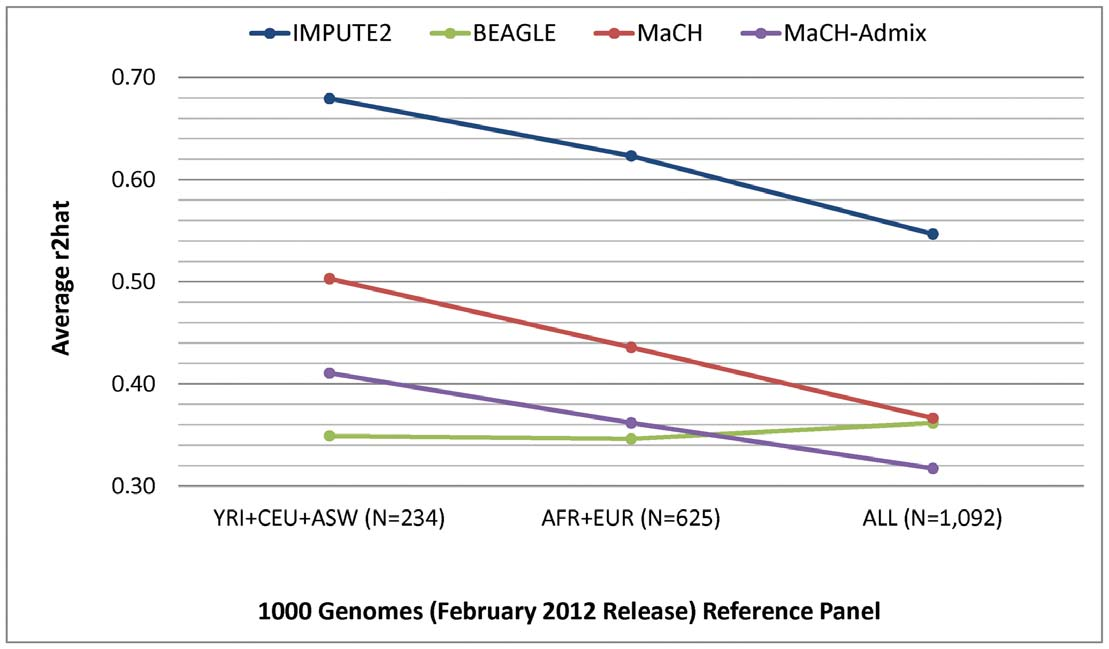
Average r2hat values resulting from four different imputation programs and three different 1000 Genomes (February 2012) reference panels. r2hat values were averaged across all imputed SNPs on chromosome 22. The number of subjects corresponding to each reference panel is shown in parentheses.

The performance results comparing the 12 imputation strategies (four different programs and three different reference panels from the February 2012 release of 1000 Genomes) are shown in Figure 1 for concordance, Figure 2 for IQS, and Figure 3 for average r2hat. Concordance and IQS served as metrics of imputation accuracy of masked genotyped SNPs, whereas average r2hat served as a metric of imputation quality of all imputed SNPs. Comparisons of the imputation performance results based on HapMap phase III and the August 2010 release of 1000 Genomes (Figures S3, S4, S5) shows that HapMap gave the best imputation performance. However, 1000 Genomes offers substantially denser coverage of the genome, and we found that the February 2012 release provided notably better imputation performance when compared to the earlier August 2010 release.

When focusing on the 1000 Genomes February 2012 release, the highest concordance rates were obtained using IMPUTE2 and MaCH, and these two programs performed equally well across the reference panels (Figure 1). Minimal differences were observed between the most closely related panel (YRI+CEU+ASW, concordance = 92.4% with MaCH and 92.3% with IMPUTE2) and the most diverse panel (ALL, concordance = 92.5% with both MaCH and IMPUTE2), despite the large discrepancy in reference sample size. Comparisons using HapMap phase III and the August 2010 release of 1000 Genomes also showed nearly indistinguishable performance for IMPUTE2 and MaCH, both of which outperformed BEAGLE and MaCH-Admix (Figure S3).

Unlike concordance, the IQS results revealed that IMPUTE2 had the highest imputation accuracy when taking MAF into account for the 1000 Genomes (February 2012 release) reference panels (Figure 2). The IQS results for the three reference panels were nearly equivalent when using IMPUTE2. As shown in Figure S4, IMPUTE2 similarly outperformed the other programs when using reference panels from the older release of 1000 Genomes. The IQS metric is particularly useful for evaluating performance of low frequency SNPs, thus suggesting that the better performance by IMPUTE2 was driven by its markedly better imputation of low frequency SNPs.

**Figure 4:**
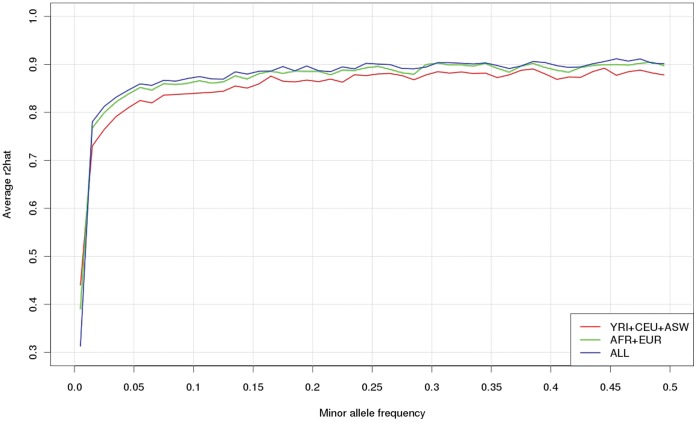
Average r2hat, based on imputation using IMPUTE2, across the minor allele frequency (MAF) spectrum. Imputation was conducted for all SNPs available on the YRI+CEU+ASW (N = 234, in red), AFR+EUR (N = 625, in green), or the ALL (N = 1,092, in blue) reference panel from 1000 Genomes. Imputed polymorphic SNPs were divided into MAF intervals of 1%, and their average r2hat values were calculated within each interval.

With regard to average r2hat, IMPUTE2 outperformed all other programs (Figure 3), but inclusion of more distantly related subjects led to reduced overall imputation quality (average r2hat = 0.68 for YRI+CEU+ASW, average r2hat = 0.62 for AFR+EUR, and average r2hat = 0.55 for ALL). The pattern of reduced overall imputation quality was entirely driven by low frequency SNPs (Figure 4), particularly SNPs with MAF≤2%, which represent 48%, 58%, and 64% of the imputed SNPs when using the YRI+CEU+ASW, AFR+EUR, and ALL panels, respectively. Inclusion of more distantly related subjects did not have the same effect on average r2hat when using the August 2010 release of 1000 Genomes (Figure S5), likely due to a smaller proportion of low frequency SNPs (e.g., 23% of imputed SNPs with MAF≤2% when based on the YRI+CEU+ASW panel and 25% when based on the AFR+EUR panel). Nonetheless, when using the February 2012 release of 1000 Genomes, imputation quality for low frequency SNPs was highest when using the most closely related reference panel (average r2hat = 0.51 when using YRI+CEU+ASW, 0.45 when using AFR+EUR, and 0.37 when using ALL, for SNPs with MAF≤2%). For SNPs in the remainder of the MAF spectrum, the highest imputation quality was observed when using the most diverse reference panel (average r2hat = 0.83 when using YRI+CEU+ASW and 0.86 when using either EUR+AFR or ALL, for SNPs with MAF>2%). This pattern resulted from poor imputation quality for low frequency SNPs in the ALL panel that are monomorphic in the more closely related panels. The pattern of reduced overall imputation quality in more diverse panels was similarly observed for the MaCH programs (Figure 3), due to poor imputation quality of low frequency SNPs (Figure S6 for MaCH and Figure S7 for MaCH-Admix). When using BEAGLE, imputation quality of low frequency SNPs was not negatively affected by the inclusion of more distantly related populations in the AFR+EUR and ALL panels (Figure 3 and Figure S8).

We evaluated two practical approaches to minimize the occurrence of low frequency SNPs that are most likely monomorphic in African Americans. First, we used the straightforward approach of imposing an r2hat threshold (e.g., the widely applied r2≥0.3 threshold) on the imputed SNP set, which reduced, but did not eliminate, the occurrence of problematic low frequency SNPs. For instance, 32.7% of the SNPs imputed based on the ALL panel were monomorphic in the YRI+CEU+ASW panel; after imposing the r2hat≥0.3 threshold, 11.0% of the remaining SNPs were monomorphic in the more closely related panel. As an alternative approach, SNPs can be filtered out based on their MAF in reference panel subpopulations (e.g., removing SNPs from the ALL reference panel that are monomorphic in the YRI+CEU+ASW panel) before or after imputation. For pre-imputation filtering, IMPUTE2 offers a filtering option (“-filt_rules_1”) that removes reference SNPs that are monomorphic in the panel of interest (e.g., YRI+CEU+ASW for African Americans), hence leaving fewer input SNP genotypes and speeding the imputation procedure. For postimputation filtering, SNPs are imputed using all available reference SNPs as input and then those that are monomorphic in the panel of interest are removed. We applied this postimputation filtering strategy to our imputation results. When considering only the 312,474 SNPs that are polymorphic across the reference panels (i.e., SNPs that are polymorphic in the YRI+CEU+ASW panel) out of 475,371 total SNPs on chromosome 22, use of the ALL panel resulted in the highest average r2hat, both overall (Figure S9 for all programs) and across the MAF spectrum (Figure S10 for IMPUTE2 specifically).

With regard to computational efficiency among the imputation programs, Howie et al. previously found that IMPUTE2 runs considerably faster and requires far less computational memory than BEAGLE [17]. Our own imputation analyses found that IMPUTE2 and MaCH-Admix were best capable of being scaled to 1000 Genomes imputations, as expected given the recent developments in these newest programs to accommodate larger reference panels while minimizing the impact on computational burden.

## Discussion

The 1000 Genomes project is being increasingly used in GWAS for SNP genotype imputation, given its highly dense SNP coverage and diverse selection of reference populations. However, there are no well-established strategies to optimize imputation performance, especially in admixed study populations. Our study extends the findings of previous studies evaluating imputation performance using 1000 Genomes reference panels [8], [29], [33], [34], by the following: (1) focusing on the more recent February 2012 release of 1000 Genomes, (2) considering the most diverse ALL reference panel, and (3) making comparisons using recently developed imputation programs for which the ALL panel are specifically advocated. We found that imputation accuracy (based on concordance and IQS results) was comparable across the reference panels. The highest overall imputation quality (based on average r2hat results) was observed for the most closely related YRI+CEU+ASW panel, but this finding was entirely driven by low frequency SNPs, most notably SNPs with MAF≤2%. More specifically, imputation quality based on the most diverse ALL panel was reduced by an abundance of population-specific SNPs that are likely absent in African Americans but present in other populations with differing MAF and linkage disequilibrium structures. The ALL reference panel had the highest overall imputation quality when these low frequency, population-specific SNPs were not considered, and this panel resulted in the highest quality for common SNPs (MAF>2%), regardless of the population specificity.

Several imputation programs are available, and our results showed that the highest genotype imputation accuracy and quality were achieved in African Americans using IMPUTE2. The IMPUTE2 program is also computationally efficient [17]. Chanda et al. [8] previously reported that IMPUTE and MaCH performed equally well in African Americans and that both programs performed consistently better than BEAGLE, regardless of the 1000 Genomes reference panel used from the August 2010 and June 2011 releases. Our comparisons of the latest IMPUTE version (i.e., IMPUTE2), BEAGLE, MaCH, and MaCH-Admix with reference to more recent 1000 Genomes panels (February 2012 release) showed the same pattern as Chanda et al. [8] when using the concordance metric to evaluate imputation accuracy. However, concordance can overestimate the agreement of imputed and true genotypes when the MAF is low, due to random chance [30]. By taking the observed agreement (i.e., concordance) and subtracting out the chance agreement derived from the SNP’s allele frequencies, IQS is less prone to overestimating the imputation accuracy of low MAF SNPs. Our calculation of the IQS metric demonstrated an advantage of using IMPUTE2 to optimize imputation accuracy, a pattern which was not apparent from the comparison of concordance rates. The average r2hat metric further demonstrated that IMPUTE2 outperformed the other imputation programs, regardless of the reference panel used. Interestingly, unlike IMPUTE2 and the MaCH programs, the overall imputation quality of BEAGLE was not negatively affected by the inclusion of more distantly related reference subjects–a pattern that might reflect the differing algorithms that underlie BEAGLE versus the IMPUTE2 and MaCH programs. BEAGLE is based on localized haplotype clusters, whereas extensions of the IMPUTE and MaCH programs are based on population genetic principles for estimating the conditional distribution of haplotypes [15].

The cosmopolitan approach of combining all available reference populations incurs more computational burden than other imputation approaches, but it has been advocated as the simplest and most practical approach without sacrificing performance [5], [6], [17], [23]. Studies focusing specifically on African American study populations have shown that inclusion of diverse reference panels is clearly advantageous over single ethnic panels [8], [28], but the optimal extent of diversity has not been fully evaluated. In European-derived study populations, Jostins et al. suggested that using diverse reference populations improved imputation of low frequency SNPs. However, their conclusion was drawn from HapMap phase III imputed SNPs that had already passed an r2 threshold of 0.9 [23]. Our study similarly showed that increasing the reference sample size by including more distantly related populations improved imputation quality, but this pattern pertained mostly to SNPs that were present in populations more closely related to African Americans. To efficiently remove the occurrence of low frequency SNPs that are most likely monomorphic in African Americans, investigators should consider filtering SNPs based on their MAF in subpopulations of interest. We found that postimputation filtering based on MAF in 1000 Genomes YRI+CEU+ASW reference subjects alleviated the problem with reduced overall imputation quality when including more distantly related reference subjects. We also found that the alternative approach of applying an r2 threshold (e.g., the widely applied r2≥0.3 threshold) was less favorable because it reduced but did not eliminate the occurrence of monomorphic SNPs in African Americans. Further, setting a stringent r2 threshold has been shown empirically to reduce statistical power, especially in regions of low linkage disequilibrium [35]. Our findings likely extend to other admixed populations. Future work is needed to evaluate the impact of using the 1000 Genomes ALL reference panel on low frequency SNPs for European-derived and other populations.

There are two potential limitations to the current study that should be considered when interpreting its results. First, we obtained genome-wide genotype data for African American study subjects from Illumina’s iControlDB, and there could be unrecognized problems in the data collection. Use of publicly available genotype data for GWAS is becoming more common as a resourceful approach to testing analytic methods (as done in our study), among other uses. Genetic studies using controls from public sources require stringent QC procedures, as demonstrated by our population structure analyses showing that several of the publicly available African American study subjects were ancestral outliers, as previously reported [36]. Second, the imputation performance patterns were deduced from chromosome 22. We compared imputation performance metrics between the largest and smallest autosomes (chromosomes 1 and 22) using the YRI reference population, and we found that imputation quality and accuracy were somewhat better on the larger chromosome 1 but not different in character from the chromosome 22 results. Other chromosomes likely have higher performance metric values than those presented here for chromosome 22, but we do not expect the genome-wide imputation patterns to differ greatly.

Our study is the first to highlight important considerations when using the cosmopolitan approach with 1000 Genomes to conduct imputation in an admixed study population, particularly an African American population. Imputation quality of all low frequency SNPs was relatively low, and imputation of low frequency, population-specific SNPs was especially prone to imputation error. Because imputation error leads to a loss of statistical power [37], [38], *p*-values of true association signals might be attenuated for the low frequency SNPs. Further, inclusion of more distantly related reference panels for imputation in African-derived study populations has been suggested to weaken association signals particularly near loci under strong selective pressure [39]. Regardless of the software program implemented, an optimal balance for African American studies may be provided by focusing GWAS analyses on SNPs imputed with reference to the ALL panel and then following up implicated regions based only on SNPs that are present in more closely related populations.

## Supporting Information

Figure S1 **Quality control procedures for African Americans genotyped on the Illumina HumanHap550v3 BeadChip from Illumina’s iControlDB.** Quality control procedures were conducted using PLINK, unless otherwise stated. At each step, the number of excluded subjects is provided. For each pair or cluster of subjects identified in steps 2 and 3, we retained only the one subject having the highest call rate.
(DOC)

Figure S2 **STRUCTURE triangle plot showing estimated ancestral proportions of African American study subjects relative to HapMap populations.** African American study subjects from iControlDB (in red) were genotyped on the Illumina HumanHap550 BeadChip version 3. Ancestral proportion estimates were based on 10,000 randomly selected HapMap SNPs in linkage equilibrium. The triangle’s vertices represent West Africans (YRI subjects in blue), European Americans (CEU subjects in yellow), and East Asians (CHB subjects in green), and the triangle’s edges indicate the ancestral proportions. African Americans from HapMap (ASW subjects in black) were also included for admixture comparison. African American study subjects with an African ancestry <60% were excluded from further analysis.
(DOC)

Figure S3 **Concordance resulting from four different imputation programs and three different reference panels from either HapMap phase III or 1000 Genomes (August 2010 release).** Concordance rates were based on masking 2% of the genotyped SNPs on chromosome 22 and comparing imputed and true genotypes. The number of subjects corresponding to each reference panel is shown in parentheses.
(DOC)

Figure S4 **Imputation quality score (IQS) resulting from four different imputation programs and three different reference panels from either HapMap phase III or 1000 Genomes (August 2010 release).** IQS results were based on masking 2% of the genotyped SNPs and adjusting the concordance rate chance agreement between imputed and true genotypes. The number of subjects corresponding to each reference panel is shown in parentheses.
(DOC)

Figure S5 **Average r2hat values resulting from four different imputation programs and three different reference panels from either HapMap phase III or 1000 Genomes (August 2010 release).** r2hat values were averaged across all imputed SNPs on chromosome 22. The number of subjects corresponding to each reference panel is shown in parentheses.
(DOC)

Figure S6 **Average r2hat, based on imputation using MaCH, across the minor allele frequency (MAF) spectrum.** Imputation was conducted for all SNPs available on the YRI+CEU+ASW (N = 234, in red), AFR+EUR (N = 625, in green), or ALL (N = 1,092, in blue) reference panels from 1000 Genomes. Imputed polymorphic SNPs were divided into MAF intervals of 1%, and their average r2hat values were calculated within each interval.
(DOC)

Figure S7 **Average r2hat, based on imputation using MaCH-Admix, across the minor allele frequency (MAF) spectrum.** Imputation was conducted for all SNPs available on the YRI+CEU+ASW (N = 234, in red), AFR+EUR (N = 625, in green), or ALL (N = 1,092, in blue) reference panels from 1000 Genomes. Imputed polymorphic SNPs were divided into MAF intervals of 1%, and their average r2hat values were calculated within each interval.
(DOC)

Figure S8 **Average r2hat, based on imputation using BEAGLE, across the minor allele frequency (MAF) spectrum.** Imputation was conducted for all SNPs available on the YRI+CEU+ASW (N = 234, in red), AFR+EUR (N = 625, in green), or ALL (N = 1,092, in blue) reference panels from 1000 Genomes. Imputed polymorphic SNPs were divided into MAF intervals of 1%, and their average r2hat values were calculated within each interval.
(DOC)

Figure S9 **Average r2hat values resulting from four different imputation programs and three different 1000 Genomes (February 2012) reference panels, considering only imputed SNPs that were polymorphic on the YRI+CEU+ASW panel.** r2hat values were averaged across the 312,474 relevant imputed SNPs out of 475,371 imputed SNPs on chromosome 22. The number of subjects corresponding to each reference panel is shown in parentheses.
(DOC)

Figure S10 **Average r2hat, based on imputation using IMPUTE2, across the minor allele frequency (MAF) spectrum.** Imputation was conducted for all SNPs available on the YRI+CEU+ASW (N = 234, in red), AFR+EUR (N = 625, in green), or ALL (N = 1,092, in blue) reference panels from 1000 Genomes, but only SNPs present across all reference panels (i.e., YRI+CEU+ASW) are shown. Imputed polymorphic SNPs were divided into MAF intervals of 1%, and their average r2hat values were calculated within each interval.
(DOC)

Table S1 **iControlDB subjects genotyped on the Illumina HumanHap550v3 BeadChip, who were identified as African American but their genetic data indicated less than 60% African ancestry.** These 179 subjects were excluded due to ancestral misclassification.
(DOC)
