# Supplementary material for: Assessment of Genotype Imputation Performance Using 1000 Genomes in African American Studies
Source: PLoS One. 2012 Nov 30;7(11):e50610. doi: 10.1371/journal.pone.0050610 (PMC3511547; doi:10.1371/journal.pone.0050610)
Supplement: Figure S1 — Quality control procedures for African Americans genotyped on the Illumina HumanHap550v3 BeadChip from Illumina’s iControlDB. Quality control procedures were conducted using PLINK, unless otherwise stated. At each step, the number of excluded subjects is provided. For each pair or cluster of subjects identified in steps 2 and 3, we retained only the one subject having the highest call rate. (DOC) [file pone.0050610.s001.doc]

1. Call rate<95%

No subjects excluded.

2) Identity-by-state estimate>90% to detect duplicate subjects.

2 subjects excluded.

3) Kinship coefficient>0.0441, using the KING program to detect cryptic relatedness.

54 subjects excluded.

4) Discordance between reported gender and chromosome X SNP data (F_ST_<0.2 indicates female and F_ST_>0.8 indicates male).

No subjects excluded.

5) Excessive homozygosity, defined by

F_ST_<-0.2 or F_ST_>0.5 based on ~230,000 autosomal SNPs in linkage equilibrium (r^2^<0.5).

No subjects excluded.

6) Ancestral misclassification, defined by pairwise population concordance P<0.0005 using PLINK [Purcell et al., 2007] or <60% African ancestry based on YRI, CEU, and CHB ancestral proportions calculated using STRUCTURE [Pritchard et al., 2000].

179 subjects excluded.

Final analysis data set: 595 subjects
