# Supplementary figures and images for: Assessment of Genotype Imputation Performance Using 1000 Genomes in African American Studies
Source: PLoS One. 2012 Nov 30;7(11):e50610. doi: 10.1371/journal.pone.0050610 (PMC3511547; doi:10.1371/journal.pone.0050610)

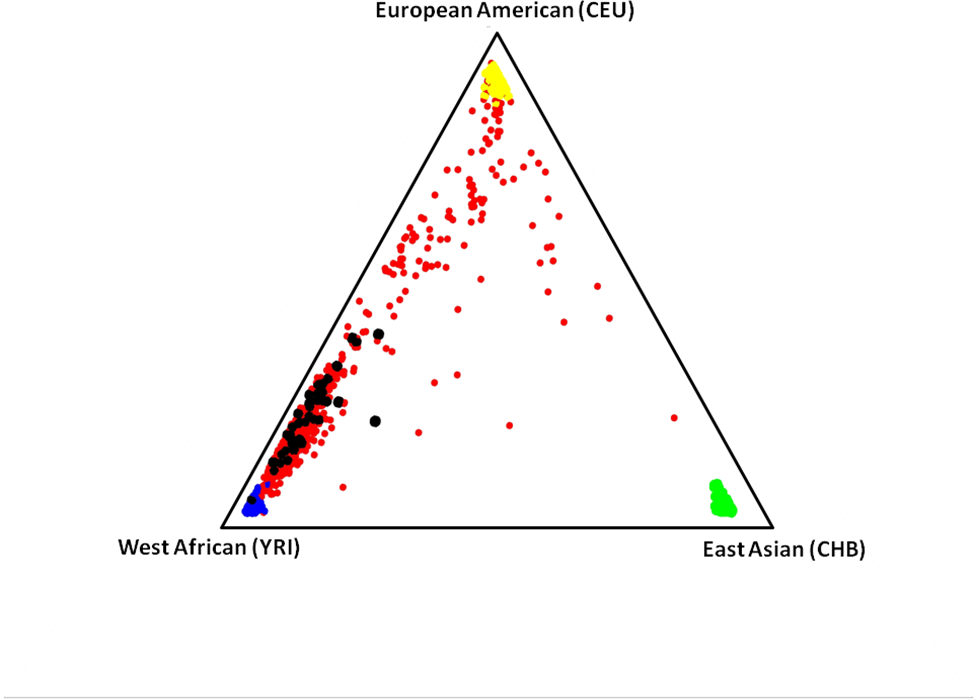

Supplement: Figure S2 — STRUCTURE triangle plot showing estimated ancestral proportions of African American study subjects relative to HapMap populations. African American study subjects from iControlDB (in red) were genotyped on the Illumina HumanHap550 BeadChip version 3. Ancestral proportion estimates were based on 10,000 randomly selected HapMap SNPs in linkage equilibrium. The triangle’s vertices represent West Africans (YRI subjects in blue), European Americans (CEU subjects in yellow), and East Asians (CHB subjects in green), and the triangle’s edges indicate the ancestral proportions. African Americans from HapMap (ASW subjects in black) were also included for admixture comparison. African American study subjects with an African ancestry <60% were excluded from further analysis. (DOC) [file pone.0050610.s002.doc]

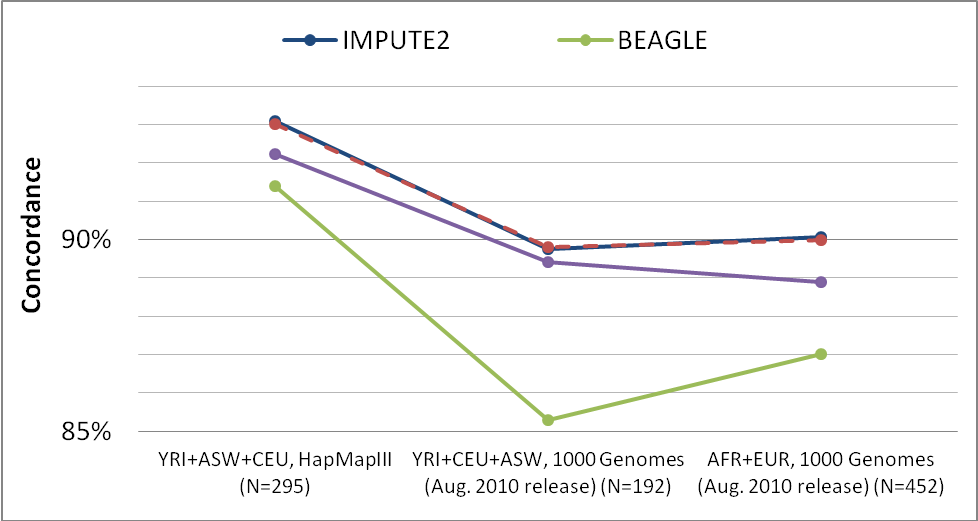

Supplement: Figure S3 — Concordance resulting from four different imputation programs and three different reference panels from either HapMap phase III or 1000 Genomes (August 2010 release). Concordance rates were based on masking 2% of the genotyped SNPs on chromosome 22 and comparing imputed and true genotypes. The number of subjects corresponding to each reference panel is shown in parentheses. (DOC) [file pone.0050610.s003.doc]

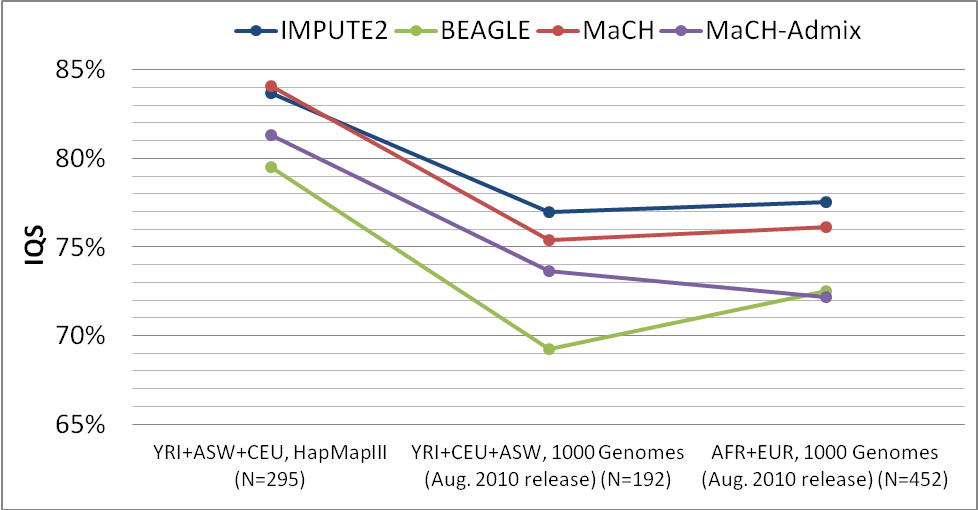

Supplement: Figure S4 — Imputation quality score (IQS) resulting from four different imputation programs and three different reference panels from either HapMap phase III or 1000 Genomes (August 2010 release). IQS results were based on masking 2% of the genotyped SNPs and adjusting the concordance rate chance agreement between imputed and true genotypes. The number of subjects corresponding to each reference panel is shown in parentheses. (DOC) [file pone.0050610.s004.doc]

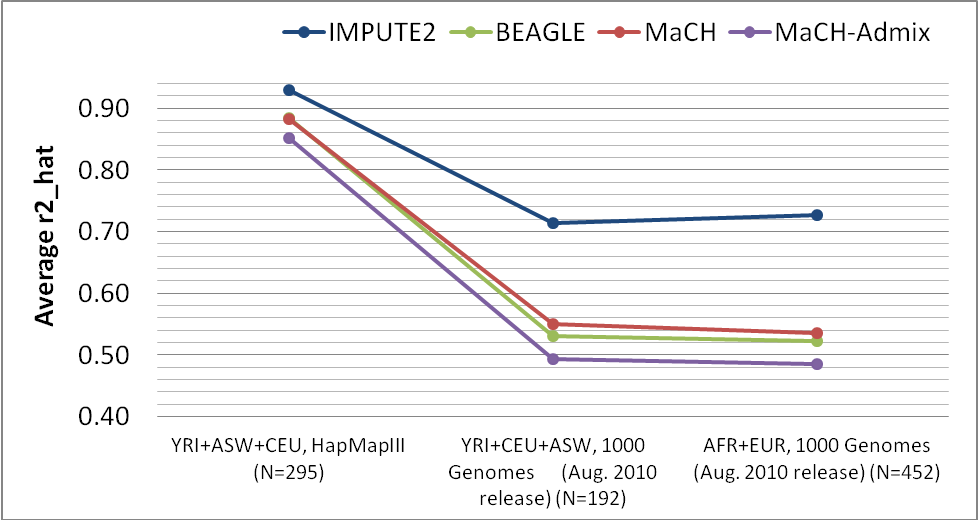

Supplement: Figure S5 — Average r2hat values resulting from four different imputation programs and three different reference panels from either HapMap phase III or 1000 Genomes (August 2010 release). r2hat values were averaged across all imputed SNPs on chromosome 22. The number of subjects corresponding to each reference panel is shown in parentheses. (DOC) [file pone.0050610.s005.doc]

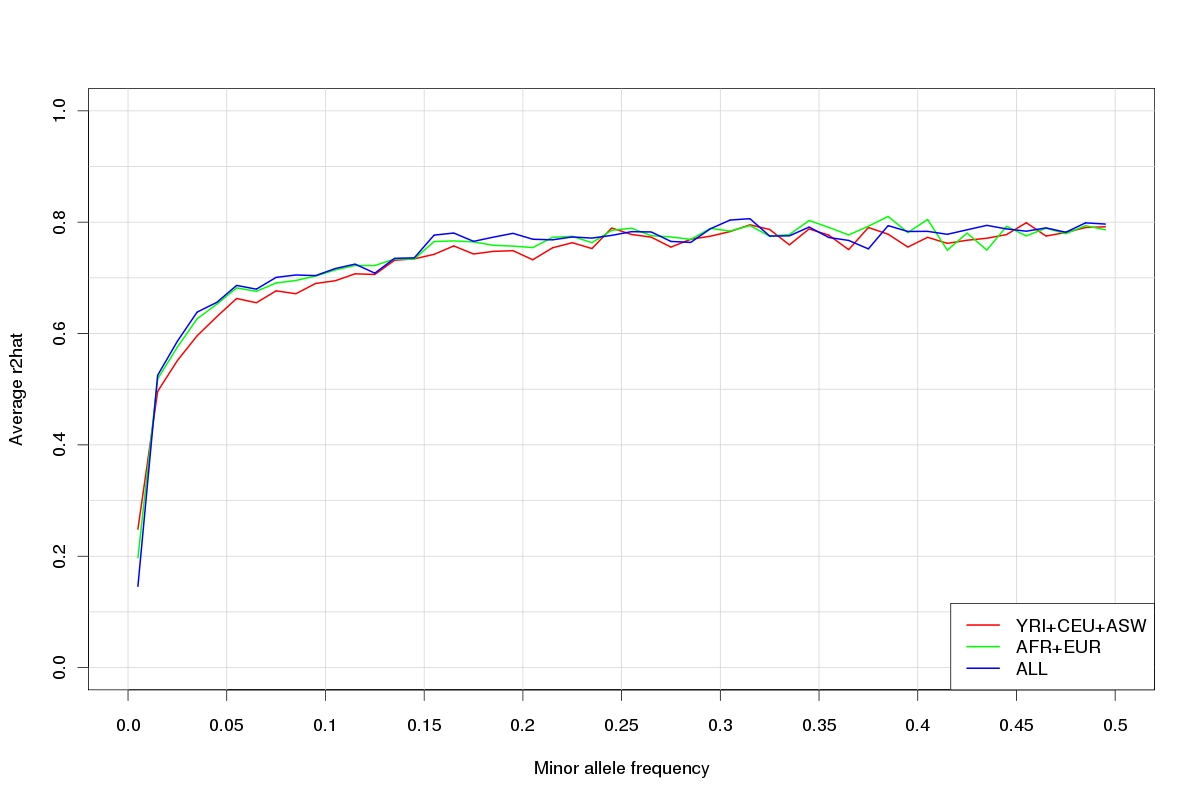

Supplement: Figure S6 — Average r2hat, based on imputation using MaCH, across the minor allele frequency (MAF) spectrum. Imputation was conducted for all SNPs available on the YRI+CEU+ASW (N = 234, in red), AFR+EUR (N = 625, in green), or ALL (N = 1,092, in blue) reference panels from 1000 Genomes. Imputed polymorphic SNPs were divided into MAF intervals of 1%, and their average r2hat values were calculated within each interval. (DOC) [file pone.0050610.s006.doc]

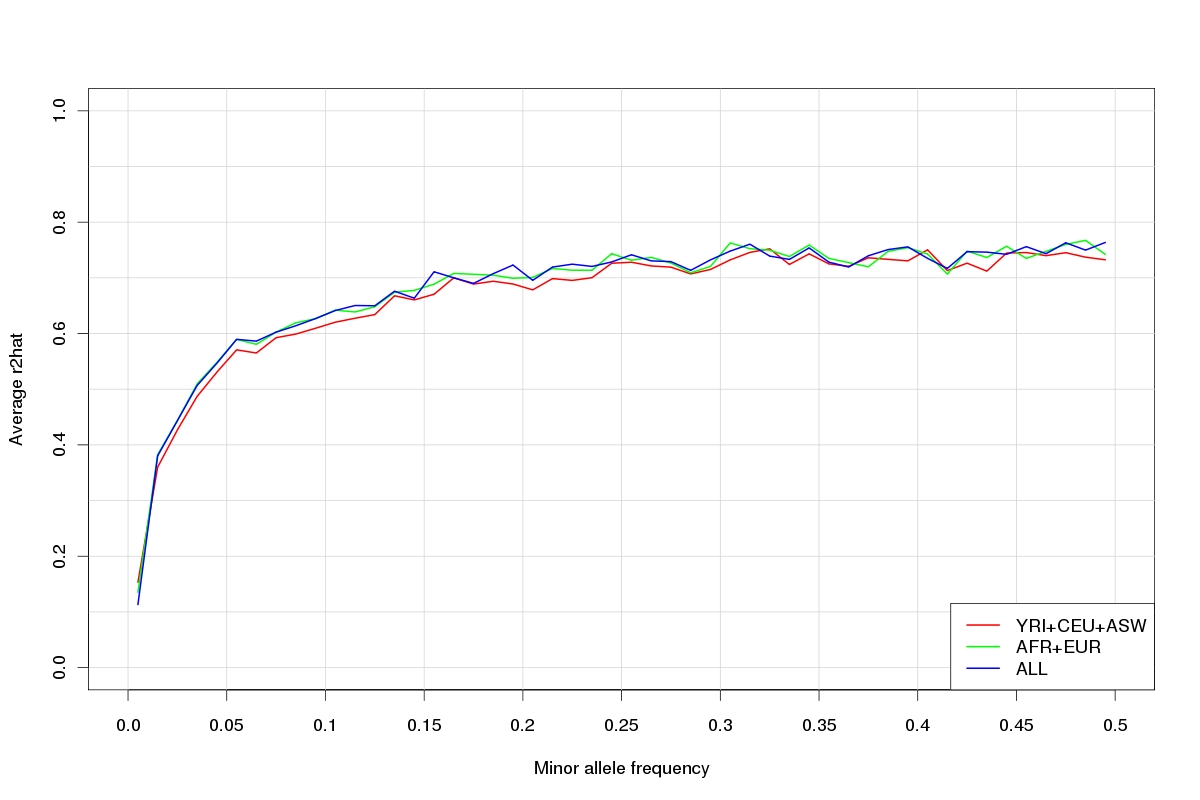

Supplement: Figure S7 — Average r2hat, based on imputation using MaCH-Admix, across the minor allele frequency (MAF) spectrum. Imputation was conducted for all SNPs available on the YRI+CEU+ASW (N = 234, in red), AFR+EUR (N = 625, in green), or ALL (N = 1,092, in blue) reference panels from 1000 Genomes. Imputed polymorphic SNPs were divided into MAF intervals of 1%, and their average r2hat values were calculated within each interval. (DOC) [file pone.0050610.s007.doc]

**
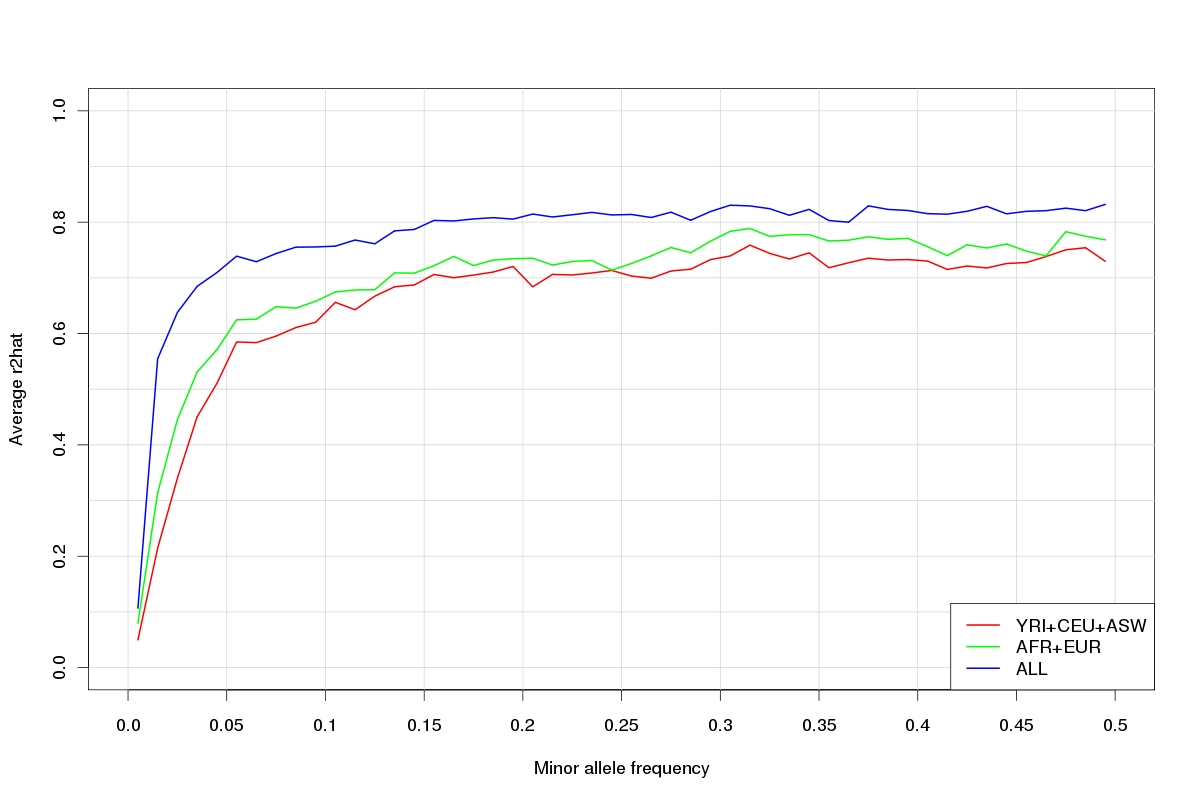
**

Supplement: Figure S8 — Average r2hat, based on imputation using BEAGLE, across the minor allele frequency (MAF) spectrum. Imputation was conducted for all SNPs available on the YRI+CEU+ASW (N = 234, in red), AFR+EUR (N = 625, in green), or ALL (N = 1,092, in blue) reference panels from 1000 Genomes. Imputed polymorphic SNPs were divided into MAF intervals of 1%, and their average r2hat values were calculated within each interval. (DOC) [file pone.0050610.s008.doc]

**
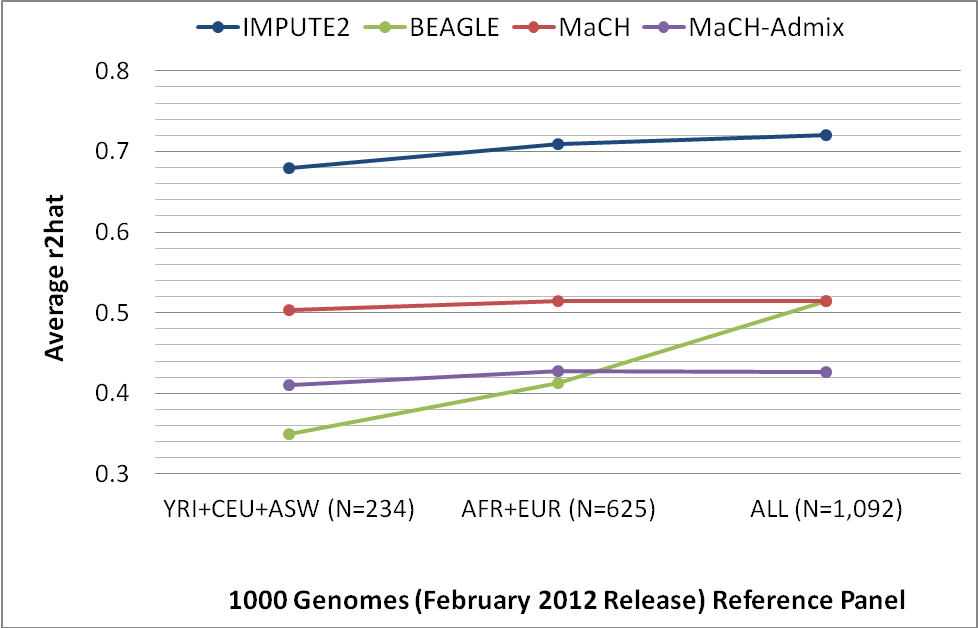
**

Supplement: Figure S9 — Average r2hat values resulting from four different imputation programs and three different 1000 Genomes (February 2012) reference panels, considering only imputed SNPs that were polymorphic on the YRI+CEU+ASW panel. r2hat values were averaged across the 312,474 relevant imputed SNPs out of 475,371 imputed SNPs on chromosome 22. The number of subjects corresponding to each reference panel is shown in parentheses. (DOC) [file pone.0050610.s009.doc]

**
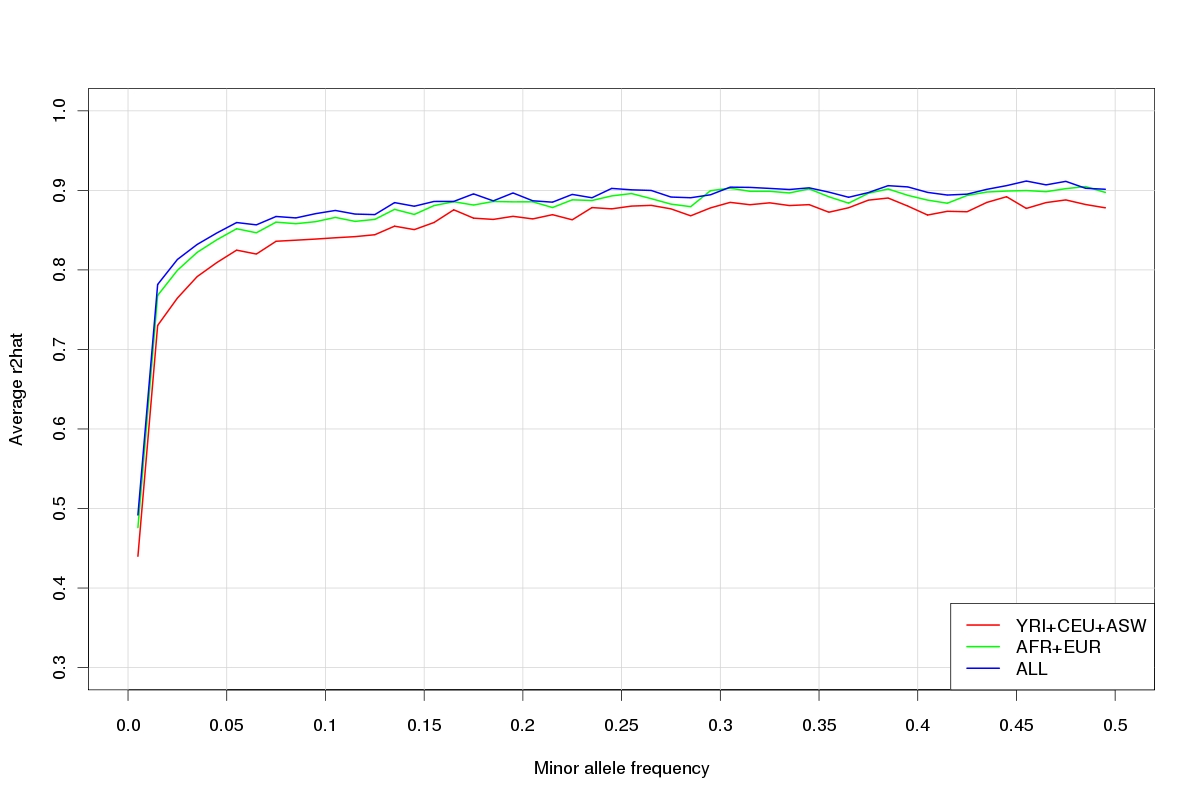
**

Supplement: Figure S10 — Average r2hat, based on imputation using IMPUTE2, across the minor allele frequency (MAF) spectrum. Imputation was conducted for all SNPs available on the YRI+CEU+ASW (N = 234, in red), AFR+EUR (N = 625, in green), or ALL (N = 1,092, in blue) reference panels from 1000 Genomes, but only SNPs present across all reference panels (i.e., YRI+CEU+ASW) are shown. Imputed polymorphic SNPs were divided into MAF intervals of 1%, and their average r2hat values were calculated within each interval. (DOC) [file pone.0050610.s010.doc]
