# Supplementary material for: Assessment of Genotype Imputation Performance Using 1000 Genomes in African American Studies
Source: PLoS One. 2012 Nov 30;7(11):e50610. doi: 10.1371/journal.pone.0050610 (PMC3511547; doi:10.1371/journal.pone.0050610)
Supplement: Table S1 — iControlDB subjects genotyped on the Illumina HumanHap550v3 BeadChip, who were identified as African American but their genetic data indicated less than 60% African ancestry. These 179 subjects were excluded due to ancestral misclassification. (DOC) [file pone.0050610.s011.doc]

| 1749718048_A | 1767676325_A | 1780846519_A | 1796039582_A | 1828951053_A |
| --- | --- | --- | --- | --- |
| 1749718112_A | 1767676347_A | 1782690003_A | 1800835257_A | 1828951077_A |
| 1749718125_A | 1767676367_A | 1782690031_A | 1800835529_A | 1832087033_A |
| 1749726001_A | 1767676389_A | 1782690061_A | 1800835535_A | 1832087096_A |
| 1749726015_A | 1767676421_A | 1782690089_A | 1803147005_A | 1832087508_A |
| 1749726020_A | 1767676434_A | 1782690113_A | 1803147044_A | 1836416437_A |
| 1749726082_A | 1767676435_A | 1782690136_A | 1803147115_A | 1836416440_A |
| 1749726195_A | 1767684232_A | 1782690162_A | 1803147162_A | 1836416598_A |
| 1749726297_A | 1767684318_A | 1782690165_A | 1803147197_A | 1838438096_A |
| 1749734320_A | 1767684366_A | 1782690211_A | 1803147206_A | 1838438132_A |
| 1759502540_A | 1767684371_A | 1782690228_A | 1803147218_A | 1838438227_A |
| 1759529359_A | 1767684430_A | 1782690257_A | 1803147266_A | 1838438242_A |
| 1759537024_A | 1767684438_A | 1782690262_A | 1813290409_A | 1838438250_A |
| 1759537025_A | 1767684567_A | 1782690295_A | 1814233055_A | 1838438339_A |
| 1759537048_A | 1773195040_A | 1782690301_A | 1814233195_A | 1838438506_A |
| 1759537085_A | 1773195198_A | 1782690402_A | 1814233198_A | 1846673612_A |
| 1759537126_A | 1773195243_A | 1782690413_A | 1814233200_A | 1848100455_A |
| 1759537194_A | 1773195246_A | 1782690430_A | 1814233515_A | 1849428022_A |
| 1759537220_A | 1773195305_A | 1782690431_A | 1814680066_A | 1849428187_A |
| 1759537261_A | 1773195389_A | 1782690432_A | 1814680083_A | 1849428213_A |
| 1759545303_A | 1773195452_A | 1788477260_A | 1814680108_A | 1849428259_A |
| 1759545347_A | 1773195581_A | 1791043031_A | 1814680197_A | 1853216126_A |
| 1759545348_A | 1780846009_A | 1791043185_A | 1814680377_A | 1853216326_A |
| 1759545424_A | 1780846038_A | 1791043231_A | 1814680396_A | 1859546877_A |
| 1759545546_A | 1780846044_A | 1791043279_A | 1814680510_A | 1862091825_A |
| 1759545555_A | 1780846092_A | 1793031078_A | 1814680593_A | 1862260020_A |
| 1759545599_A | 1780846105_A | 1793031088_A | 1815228134_A | 1867482859_A |
| 1765241086_A | 1780846129_A | 1793031155_A | 1815228293_A | 1867482863_A |
| 1765241098_A | 1780846270_A | 1793031276_A | 1815228362_A | 1870075704_A |
| 1765241106_A | 1780846306_A | 1793031301_A | 1815228490_A | 1870075752_A |
| 1765241138_A | 1780846313_A | 1793031355_A | 1820684044_A | 1870147682_A |
| 1765241140_A | 1780846344_A | 1793031387_A | 1820684175_A | 1871852148_A |
| 1765241185_A | 1780846356_A | 1796039092_A | 1820684177_A | 1873031637_A |
| 1765241186_A | 1780846407_A | 1796039523_A | 1820684537_A | 1873031712_A |
| 1765259219_A | 1780846412_A | 1796039534_A | 1825426145_A | 1873031851_A |
| 1767676265_A | 1780846504_A | 1796039552_A | 1825426378_A |  |
